# Minor Shoulder Injury Reveals Spontaneous Regression of Proximal Humerus Osteochondroma

**DOI:** 10.7759/cureus.16793

**Published:** 2021-07-31

**Authors:** George Kalifis, Theodorakys Marin Fermin, Efstathios Konstantinou, Vasilios Raoulis, Michael Hantes

**Affiliations:** 1 Department of Orthopedic Surgery & Musculoskeletal Trauma, University General Hospital of Larissa, Larissa, GRC; 2 Department of Traumatology, Hospital Universitario Periférico de Coche, Caracas, VEN; 3 Emergency Department, University General Hospital of Larissa, Larissa, GRC

**Keywords:** osteochondroma, spontaneous regression of osteochondroma, benign tumor shoulder, paediatric orthopedics, shoulder tumor

## Abstract

Osteochondromas are the most common benign bone tumor; nonetheless, the natural history is poorly understood as a result of the low threshold for resection and the fact that many of these lesions are asymptomatic and therefore never diagnosed. We present a case of a 17-year-old patient whose routine shoulder X-ray evaluation, due to a minor shoulder injury, revealed spontaneous regression of a previously documented left proximal humerus osteochondroma at six years follow-up. The likelihood of spontaneous regression should be better understood by orthopedic surgeons and taken into account in the decision process of whether to remove osteochondromas surgically or wait.

## Introduction

Osteochondromas are bone tumors covered by cartilage, most frequently arising from the metaphyseal bone. The vast majority of these lesions are incidental findings, comprising 35% of all primary benign bone tumors [1]. Approximately 85% of osteochondromas are solitary lesions, while nearly 15% appear as multiple hereditary osteochondromas [2]. Despite being innocent, symptomatic lesions presenting with pain, neurovascular involvement, rapid progression, and malignant transformation may benefit from surgical intervention [1-3].

Nevertheless, the literature is scarce regarding the natural history of solitary osteochondroma [4]. Recent studies evaluating long-term outcomes of conservative management of these lesions suggest that spontaneous regression of solitary osteochondromas may not be as rare as previously thought [4-6].

We present a case of a 17-year-old patient whose routine shoulder X-ray evaluation, due to a minor shoulder injury, revealed spontaneous regression of a previously documented left proximal humerus osteochondroma at six years follow-up.

## Case presentation

A 17-year-old male patient presented to our Emergency Department after two weeks of mild anterior shoulder ache of insidious onset, without a history of trauma, exacerbated by intense physical activity. Although the patient was barely symptomatic, he insisted on attending the Emergency Department being worried due to a history of a solitary osteochondroma of the left proximal humerus at the age of 11 years, which was decided to be left untreated.

**Figure 1:**
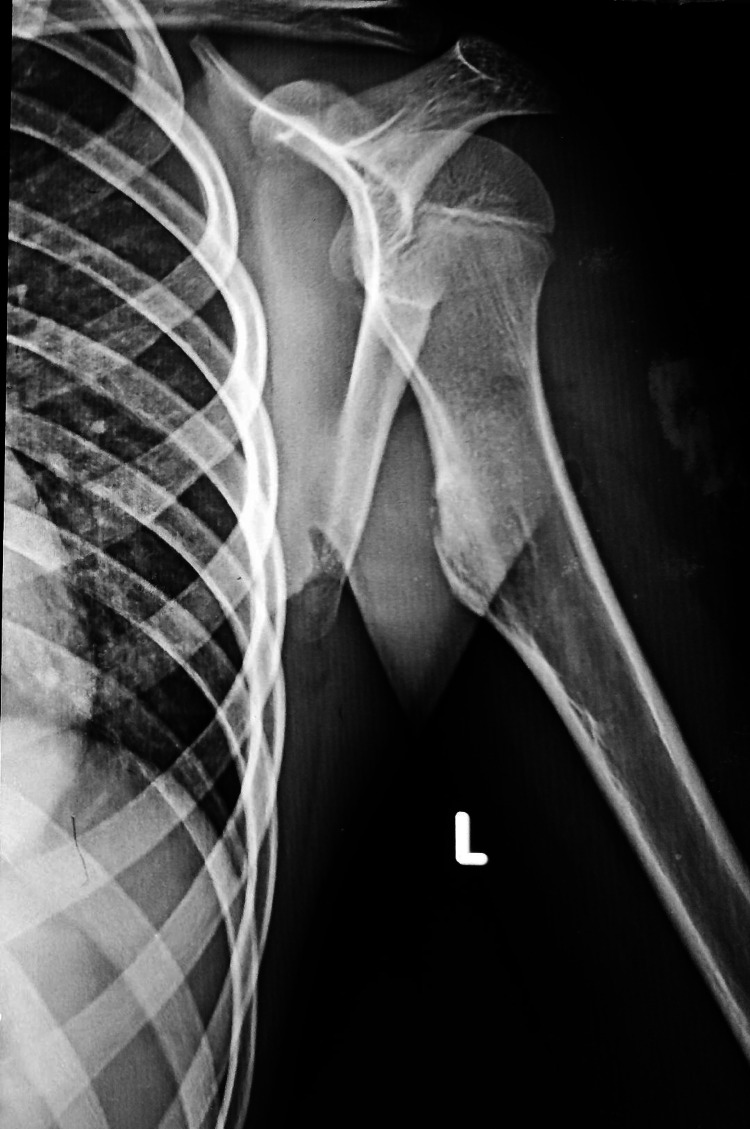
Left shoulder X-ray showing the presence of a sessile osteochondroma of the left proximal humerus at 11 years of age.

Six years before, in March 2015, the patient was referred to our department after experiencing anterior shoulder pain for a month, mainly related to physical activity. A solid, tender, immobile palpable mass of the upper third of the anteromedial arm was found and documented. Absence of previous trauma, weight loss, night fever, or any other symptoms or signs suggesting malignancy were noted. Blood tests did not reveal any abnormality. The patient’s neurovascular status was intact. The family history and past medical history of the patient were not contributory. X-ray evaluation showed a solitary sessile osteochondroma of the left proximal humerus (Figure 1).

**Figure 2:**
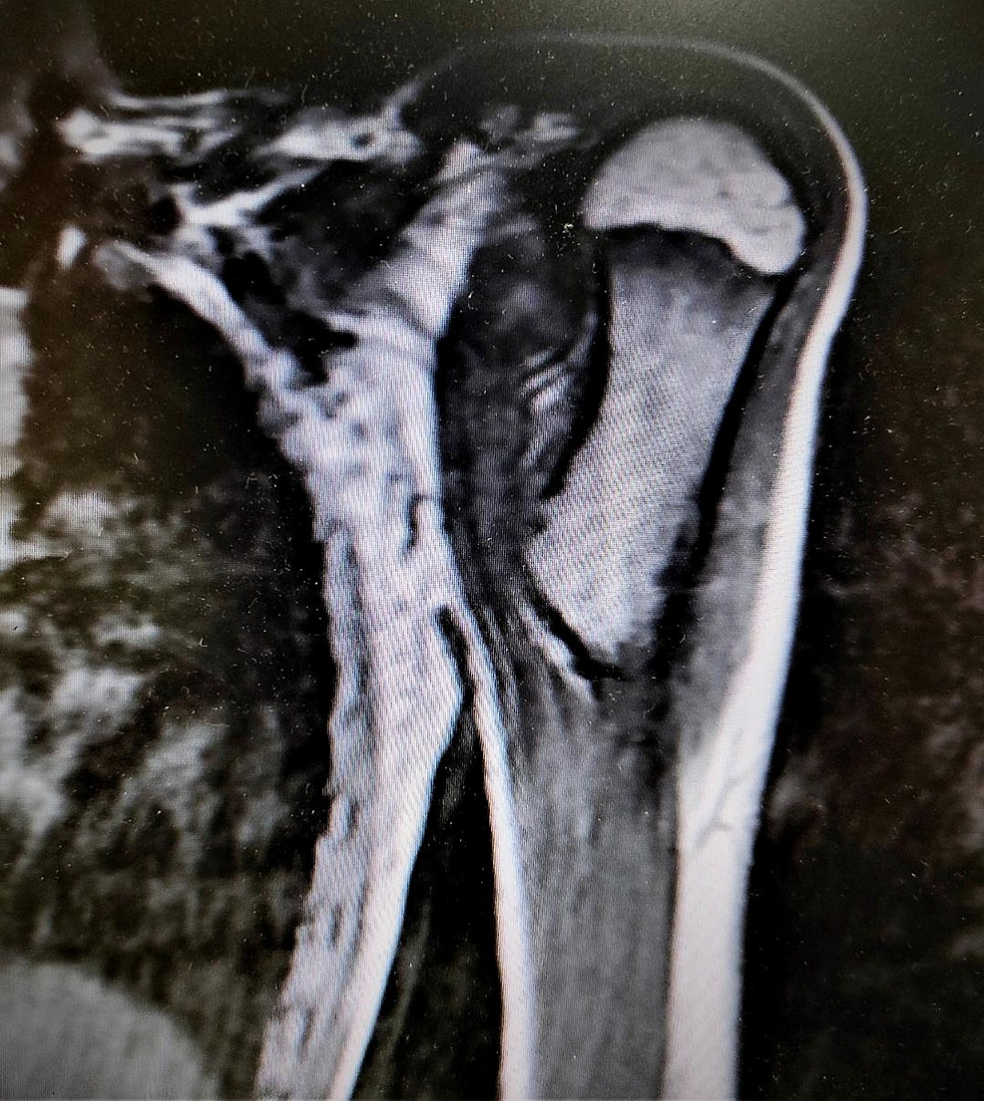
Left shoulder MRI showing a 4-cm bony protuberance covered by a 4-mm cartilage cap (osteochondroma).

Magnetic resonance imaging (MRI) examination revealed a 4-cm triangular bone protuberance surrounded by a 4-mm cartilage cap, without enhancement after intravenous contrast agents administration (Figure 2).

According to the Radiology Department's report, the lesion had features of an osteochondroma without malignant characteristics. However, imaging could not rule out a primary malignant bone tumor. The thorough clinical and radiographic evaluation did not show multiple hereditary exostoses. After explicitly discussing the treatment options with the patient and his family, an excisional biopsy was suggested. However, the patient's family refused to proceed to any intervention and did not return for follow-up.

**Figure 3:**
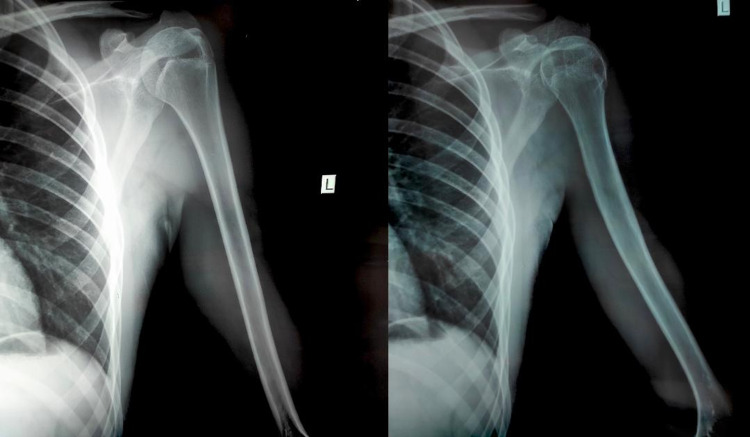
A routine shoulder X-ray due to minor injury shows incidental spontaneous regression of the proximal humerus osteochondroma at a six-year follow-up.

During his Emergency Department attendance, physical examination of the left shoulder showed full range of motion (Flexion = 180°, Abduction = 180°, Extension = 40°, Internal rotation = 70°, External rotation = 90°), equal to the uninvolved limb, without evidence of shoulder girdle muscle weakness (Muscle power 5/5). Functional assessment using patient reported outcome measures (PROMs) took place. Constant shoulder score stood at 96/100, Oxford shoulder score stood at 46/48. Also, the palpable mass that was documented during the past examination was not present. The patient’s full neurovascular assessment was normal as well. The patient also mentioned that he is a recreational basketball player who started playing basketball three years ago without any limitations regarding his left shoulder. Surprisingly, plain X-rays of the left proximal humerus were normal (Figure 3).

The previously documented osteochondroma had spontaneously regressed, and total remodeling of the bone was observed.

## Discussion

The current case report presents the spontaneous regression of a left proximal humerus osteochondroma within six years of its initial diagnosis with plain radiographs and MRI confirmation. The most important feature of this case report is its late regression, as less than 1% of these rare entity regressions occur after 15 to 19 years of age [4].

The regression of solitary osteochondromas is a rare entity [3]. Many theories have been proposed to explain these regressions, like active resorption or metaphyseal remodeling [3-4,7-8]. In the present case, the progressive incorporation of the lesion in the cortex by positional growth of the adjacent bone seems the most plausible explanation.

The proximal humerus and the distal femur have the most reported regions of spontaneous regression of osteochondromas in the literature, presumably because of the higher incidence in these regions [3,4,8]. Τhe lesions can appear either sessile or pedunculated, and some authors have theorized that it may play a role in the time for regression [4,8]. Claikens et al. reviewed 10 cases of resolving osteochondroma reported in the literature, seven were sessile, and three were pedunculated [9]. The age at diagnosis was limited to a range from 5 to 11 years, and resolution occurred before skeletal maturity was reached or within six years of initial diagnosis [3,9].

A full-body examination is essential during the initial diagnosis to rule out multiple osteochondromas, especially in children up to 12 to 14 years of age [1]. The essential diagnostic tool is a plain radiograph, but an MRI examination of the affected area is critical for differential diagnosis and to predict the likelihood of malignancy [4]. Solitary, asymptomatic, and non-operated upper and lower limb lesions should be monitored using regular self-exam, clinical and radiographic control every two to three years. If growing of the lesion is noticed or MRI findings are unclear, biopsy or excision is indicated [10].

Osteochondroma excision, like any other surgical procedure, is not exempt from complications. Wirganowicz and Watts in a retrospective case series of 80 patients with 285 lesions, found a complication rate of a 12.5% in solitary osteochondromas. Peroneal neurapraxia was the most commonly observed complication (17.5%) [11]. Other complications accounted for less than 1% and included arterial laceration, compartment syndrome, and fibular fracture.

An expectant approach of the asymptomatic or incidental lesions should be followed, especially in solitary sessile lesions up to the second decade of living, and when symptomatic, treated accordingly [4,8]. Education of the patient and family may play an essential role in managing these lesions, reducing the need for and risks of surgery [4]. Although the report of regressions in these lesions remains infrequent, the lack of a proper incidence limits the strength of any recommendation.

The present case report is adding valuable information regarding natural history and management of humerus osteochondromas. In this case the patient denied undergoing surgical excision and lost follow-up. Consequently, spontaneous regression was the outcome of non-proposed conservative treatment at a six-year follow-up. Educationally, our case may prove helpful because: The final outcome (although incidental) was good despite the initial suggestion of the surgeon; may highlight the role of conservative treatment of proximal humerus osteochondromas at a long-term follow-up; may provide useful data for further evidence-based research.

A limitation to this study is the lack of histopathological confirmation, but the MRI characterization of the present lesion was strong enough to support the diagnosis. Another limitation was the lack of full body examination.

## Conclusions

In conclusion, regression of solitary osteochondromas remains under-reported and under-diagnosed. The expectant approach is advisable during the first two decades of living, as spontaneous regression may still be feasible.
